# Preventive Effect of Naringin on Metabolic Syndrome and Its Mechanism of Action: A Systematic Review

**DOI:** 10.1155/2019/9752826

**Published:** 2019-02-03

**Authors:** Sivanesan Raja Kumar, Elvy Suhana Mohd Ramli, Nurul Alimah Abdul Nasir, Nafeeza Hj Mohd Ismail, Nur Azlina Mohd Fahami

**Affiliations:** ^1^Department of Pharmacology, Faculty of Medicine, Universiti Kebangsaan Malaysia, 56000 Batu 9 Cheras, Kuala Lumpur, Malaysia; ^2^Department of Anatomy, Faculty of Medicine, Universiti Kebangsaan Malaysia, 56000 Batu 9 Cheras, Kuala Lumpur, Malaysia; ^3^Department of Pharmacology, Faculty of Medicine, Universiti Teknologi Mara, 47000 Sungai Buloh, Selangor, Malaysia; ^4^Dean, School of Medicine, International Medical University, 57000 Bukit Jalil, Kuala Lumpur, Malaysia

## Abstract

**Background:**

Metabolic syndrome (MetS), which consists of cluster of conditions, hypertension, hyperlipidemia, hyperglycemia, and visceral obesity, is affecting population worldwide. Studies have shown that plant derived flavonoids have the ability to alleviate MetS. Naringin is a type of glycoside flavonoid found in most plant and it plays a critical role in the treatment of MetS due to its antioxidant activity and ability to regulate cytokines.

**Methods:**

A systematic review was done to study the effects of naringin on the metabolic diseases using electronic databases which include Ovid and Scopus using specific descriptors published from the year 2010 till present to provide updated literature on this field. The articles were assessed and chosen based on the criteria in which the mechanisms and effects of naringin on different metabolic diseases were reported.

**Results:**

Thirty-four articles were identified which referred to the studies that correspond to the previously stated criteria. Subsequently after screening for the articles that were published after the year 2010, finally, 19 articles were selected and assessed accordingly. Based on the assessment, naringin could alleviate MetS by reducing visceral obesity, blood glucose, blood pressure, and lipid profile and regulating cytokines.

**Conclusions:**

Naringin is an antioxidant that appears to be efficacious in alleviating MetS by preventing oxidative damage and proinflammatory cytokine release. However, the dosage used in animal studies might not be achieved in human trials. Thus, adequate investigation needs to be conducted to confirm naringin's effects on humans.

## 1. Introduction

Metabolic syndrome (MetS), previously described as Insulin Resistance Syndrome, Deadly Quartet, and Syndrome X, is becoming increasingly common among individuals around the globe. MetS encompasses a variety of risk factors which include raised blood pressure (hypertension), raised lipids level (hyperlipidemia), raised fasting glucose level (hyperglycemia), and abdominal obesity [1, 2]. The surge in the prevalence of the attributable components of MetS is causing a worldwide pandemic with implications for both clinical and public health [3]. According to International Diabetes Federation in 2006 [4], an estimation of 20-25% of the world's adult population is affected by metabolic syndrome. MetS is a major contributing factor for the development of type 2 diabetes and cardiovascular diseases [4]. MetS is caused by excessive dietary intake and the lack of physical activities that can be controlled over time [5]. However, other risk factors such as age, ethnicity, and family history could not be controlled over time [5].

Pharmacological medications such as angiotensin converting enzyme (ACE) inhibitor for hypertension, statins for hyperlipidemia, metformin, and glybenclamide for hyperglycemia have been successfully developed and used for the treatment of each component in Mets [6]. Tragically, due to various side effects, adverse reaction, and huge cost of the medications, the current drug developments are focusing more on using bioactive compounds from plants as they are relatively cheap and considered safe to be consumed without undesirable side effects [7].The phytonutrients in the plants and fruits such as flavonoids, anthocyanins, tannins, and phenolic acids are responsible for their medicinal effects on range of diseases from headaches to metabolic syndrome and cancer [7].

Flavonoid is a type of bioactive compound found in plant in substantial proportion. Over 5000 flavonoids have been identified from various plants which are classified into several groups known as anthoxanthins, flavanones, flavanonols, flavans, and flavones [8]. Studies have shown that these flavonoids are responsible for a variety of pharmacological effects due to their high antioxidant activity in both* in vivo* and* in vitro* systems [9]. The antioxidant activity action includes suppression of reactive oxygen species (ROS) formation by either inhibition of enzymes or chelating trace elements involved in free radical generation [10, 11]. As flavonoid could not be produced by our body system, it had to be taken through diet.

Naringin (4',5,7-trihydroxy flavonone-7-rhamnoglucoside) is a predominant flavanone glycoside (flavonoid) found in many plants mainly citrus fruits [12]. Naringin has been reported to show various pharmacological benefits such as antioxidant, antimicrobial, anti-inflammatory, antiapoptotic, and antimutagenic activities [13]. Apart from that, it has been proven to have no side effects on the studied models [14]. In addition, the significance of naringin has earned considerable attention of its use as cholesterol-lowering and anti-atherogenic agent in previous animal studies. With this background, the present systematic review was carried out to investigate the preventive effects of naringin and their mechanism of action in the context of obesity and metabolic syndrome.

## 2. Methodology

### 2.1. Literature Search Strategy

This systematic review was designed and carried out through a literature search conducted from May 13, 2017, to September 20, 2017, using articles and papers from databases such as Ovid and Scopus. This search strategy was done using a combination of the following sets of keywords (1) naringin OR flavanone AND (2) hypertension OR hyperlipidemia OR hyperglycemia OR dyslipidemia OR metabolic syndrome OR blood pressure OR blood glucose OR obesity. The reference lists from the extracted reports were hand searched for potential additional studies.

### 2.2. Inclusion and Exclusion Criteria

The articles selection was based on the inclusion criteria: Articles were included if they meet the following criteria: (1) original papers with independent data (2) reporting on the mechanism and effect of naringin on hyperlipidemia, hyperglycaemia, hypertension, and obesity (3) articles from 2007 to 2017 and (4) articles written in English from any country. Articles were excluded if they were (1) review articles, (2) articles written in other language, (3) addition of another compound together with naringin (4) articles published before the year 2007, and (5) duplicate studies.

### 2.3. Data Extraction

Two authors independently screened the titles and abstracts of each eligible study. From the selected publications, the following data were extracted: sample size; sample type; year of study; dosage used; duration of treatment period; outcomes observed. Disparities were resolved through final discussion. The outcomes on effect of naringin on Mets were reported by measuring fasting total low-density lipoprotein (LDL), high density lipoprotein (HDL), triglycerides (TG), total cholesterol (TC); systolic, diastolic, and mean arterial pressure; fasting glucose and insulin; oral glucose tolerance; waist circumference and body weight.

## 3. Results

### 3.1. Selection of Articles

**Figure 1:**
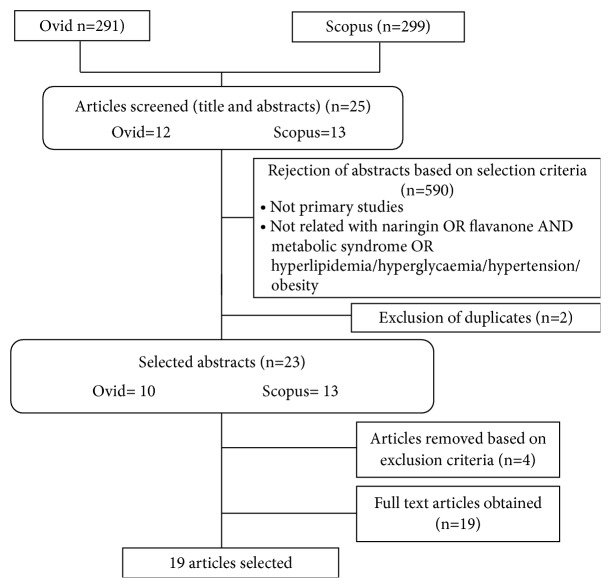
Flow diagram of study selection.

A total of 590 articles were identified from the following databases: 291 from Ovid and 299 from Scopus. Two authors independently screened the resulted articles based on title and abstract. Out of this total, 25 articles, 12 from Ovid and 13 from Scopus, were retrieved for further assessment because they were related to naringin and metabolic diseases. 2 duplicate articles listed in two or more databases were removed as it was considered only one. Moreover, 23 articles were chosen after excluding the duplicates and articles that did not match the inclusion and exclusion criteria was excluded. The chosen articles were retrieved between 2007 and 2017 as they were considered newest articles within a decade. Finally, a total of 19 articles were chosen for this systematic review as they fulfilled the inclusion and the exclusion criteria. A flowchart was created to represent the study selection as shown in Figure 1.

### 3.2. Study Characteristics

Based on the selected articles from 2007 to 2017, 2 articles reported on obesity parameters, 5 articles on the proinflammatory cytokines, 10 articles on oxidative stress, 7 articles on hyperlipidemia, 10 articles on hyperglycaemia, and 3 articles on hypertension. Together with the Mets parameters, other parameters were also extracted from the chosen studies. The chosen articles consist of only animal studies where 16 of them used rats, 2 studies used mice, and 1 study used lambs. In addition to that,* in vitro* antioxidant study using dipeptidyl peptidase-4 (DPP-IV) and* in silico* hydrogen docking study using Molegro Virtual Docker 4.1.0 trial version studies were also included. All the outcome of the naringin treated group was compared with a control group or placebo. Apart from that, all the studies reported that naringin as a pure compound was purchased through external suppliers and each study used naringin at different dosage and treatment period on its effect on Mets. In most studies reported, streptozotocin (STZ) Mets model and high fat diet (HFD) were administered to the animals in order to develop Mets. A summary table was created to represent the effect of naringin on Mets as shown in Table 1.

### 3.3. Effect of Naringin on Obesity

Naringin treated rats for 8 weeks at a dosage of 95.4±2.2 mg/kg/day reduced the abdominal fat deposition and attenuated the increase abdominal circumference [7]. After treatment with 0.2 g/kg naringin for 10 weeks, reduction in the body weight, liver weight, Lee's index, and visceral fat were observed in high diet fed mice [15].

### 3.4. Effect of Naringin on Hyperlipidemia

Rats that are treated with naringin at a dosage of 50 mg/kg for 45 days lowered plasma low-density lipoprotein (LDL) concentrations and increased plasma high-density lipoprotein (HDL) concentration significantly compared with the nontreated diabetic rats. In addition, the hepatic triglyceride and cholesterol levels were also significantly reduced with the treatment of naringin compared to the nondiabetic rats. Apart from that, the enzyme activity of HMG-CoA reductase and Acyl-CoA cholesterol acyltransferase (ACAT) were significantly reduced compared to the diabetic rats [16]. Naringin supplementation of 100mg to high fat diet fed rats for 8 weeks significantly increased both total cholesterol and triglyceride liver contents without causing any hepatocyte damage [17]. Naringin supplementation of 1.5 g/kg for 7 weeks showed significant reduction in the plasma triglyceride and cholesterol levels compared to the control lambs [18]. Plasma total cholesterol, triglyceride and nonessential fatty acid (NEFA) levels of the naringin treated rats group were reduced after 8 weeks. Naringin treatment also normalised liver wet weight and reduced deposition of fat droplets in liver [7]. Naringin at a dosage of 50 and 100 mg/kg for 28 days significantly decreased total cholesterol, triglyceride, and LDL levels and increased HDL level in diabetic rats [19]. Naringin treatment at the dosage of 0.2 g/kg for 10 weeks reduced the cholesterol and LDL levels but increased the HDL levels of high-fed diet mice. However, there was no significant change in the triglyceride level as compared to the control group mice [15]. The administration of naringin at dosage of 50 mg/kg for 30 days ameliorated serum cholesterol, triglycerides, LDL, very low-density lipoprotein (VLDL), and free fatty acid and significantly increased the HDL cholesterol in diabetic rats. Furthermore, the liver HMG-CoA reductase activity was significantly decreased as a result of naringin treatment [20].

### 3.5. Effect of Naringin on Hyperglycemia

Naringin treatment of dosage 40 mg/kg twice daily for 10 days significantly reduced serum dipeptidyl peptidase-4 (DPP-IV) enzyme activity and random glucose concentrations with increased in insulins levels in albino male rats. However, nonsignificant decrease was observed in the fasting glucose concentrations [21]. Naringin at dosage of 50 and 100 mg/kg for 28 days corrected impaired glucose utilization and insulin insensitivity in diabetic rats. Furthermore, naringin at 100 mg/kg for 28 days improved the reduced *β*-cell function in diabetic rats [19]. Naringin supplementation at dosage of 50 mg/kg for 4 weeks significantly improved the elevated blood glycosylated haemoglobin (HbA1_C_) in diabetic rats. However, the decreased serum insulin level was significantly increased in diabetic rats as a result of naringin treatment. In addition, treatment with 50 mg/kg naringin for 4 weeks had improvement in the elevated oral glucose tolerance in diabetic rats [22]. The elevated oral glucose tolerance test (OGTT) and HbA1c in diabetic rats significantly improved with the treatment of naringin at a dosage of 50 mg/kg for 30 days. In addition, the decreased serum insulin level was increased as a result of the naringin treatment at a dosage of 50 mg/kg for 30 days [20]. At a dosage of 50 mg/kg for 56 days, naringin significantly decreased the elevated fasting blood glucose, significantly improved plasma insulin, and also significantly increased hepatic glycogen content but did not improve OGTT levels in diabetic rats [23]. The elevated HOMA-IR index (assessment for insulin resistance) in the high diet fed mice was decreased by 20.3% as a result of treatment with naringin with dosage 0.2 g/kg for 10 weeks. In addition to that, there was a significant decrease in the fasting blood glucose and serum insulin levels as a result of naringin treatment at a dosage of 0.2g/kg for 10 weeks [15]. The fasting blood insulin and hepatic glycogen levels of the STZ-induced rats were significantly improved with the treatment with 50 mg/kg of naringin for 42 days [24]. Naringin treatment of 50 mg/kg for 56 days significantly reduced polydipsia compared to the untreated diabetic rats but did not significantly change in the level of fasting blood glucose [25]. The treated rats with naringin at 50 mg/kg for 56 days significantly reduced fasting blood glucose and increased plasma insulin concentrations [26]. Diabetic rats treated with naringin at dosage of 80 mg/kg BW for 42 days significantly retained the elevated levels of blood glucose and reduced levels of plasma insulin [27].

### 3.6. Effect of Naringin on Hypertension

The treatment with naringin at a dosage of 40 mg/kg showed an elevation in systolic and diastolic blood pressure at 15, 30, 45, 60, and 90 minutes compared to sham rats. In addition, naringin at a dosage of 80 mg/kg for 4 weeks showed significant antihypertensive potential by reinstating systolic and diastolic blood pressure at 15, 30, 45, 60, and 90 minutes compared to sham rats. Furthermore, the increase in mean arterial blood pressure was lessened with the treatment of naringin 80 mg/kg at 15, 30, 45, 60, and 90 minutes compared to sham rats [28]. Naringin at a dosage of (250, 500, and 1000 mg/kg) significantly suppressed the increase in systolic blood pressures after treatment for 4 weeks in spontaneously hypertensive rats [29]. The elevated systolic blood pressure in the high diet fed rats was reduced with the treatment of naringin at dosage of 100 mg/kg for 8 weeks [7].

### 3.7. Effect of Naringin on Oxidative Stress

Naringin treatment at dosage of 2 mg/kg significantly raised the glutathione-s-transferase (GST), catalase (CAT), superoxide dismutase (SOD), and glutathione (GSH) concentrations and was accompanied by significant reduction of lipid peroxidation in doxorubicin induced oxidative stress rats [30]. Naringin at dosage of 0.2 g/mg for 10 weeks provides protection from oxidative stress by increasing the activity SOD, glutathione peroxidase (GSH-Px), CAT, GSH, total antioxidant capacity (T-AOC), and decreasing the activity of malondialdehyde (MDA) in high-fat diet mice [15]. Rats treated with 10 mg/kg of naringin for 46 days decreased the MDA levels and increased the levels of SOD and catalase in diabetic rats [31]. Naringin treated diabetic rats at a dosage of 50 mg/kg for 42 days significantly ameliorated serum and hepatic MDA, nitric oxide (NO), and GSH levels, and reduced the elevated levels of MDA in diabetic rats [24]. The SOD activity in the naringin treated group at dosage 50 mg/kg which was for 56 days significantly increased by 65% compared to untreated diabetic rats [25]. Naringin at dosage of 50 mg/kg for 56 days significantly reduced plasma and cardiac MDA concentration by 71% and 60% respectively in diabetic rats. Moreover, naringin also significantly reduced plasma and cardiac protein carbonyls, respectively, in diabetic rats. The glutathione peroxidase (GPx) activity of diabetic rats was significantly reduced and the SOD activity was significantly increased with the treatment of naringin at a dosage of 50g/kg for 56 days [26]. The elevated MDA concentration in the kidney of the diabetic rats was significantly reduced and SOD concentrations was significantly increased by naringin treatment at dosage 50 mg/kg for 56 days [23]. A decrease in the levels of TBARS and increase in the activities of SOD and GSH-Px were observed in serum, pancreas, liver, and kidney of the naringin treated diabetic rats at a dosage of 50 and 100mg/kg for 28 days [19]. Naringin treated with diabetic rats at a dosage of 80mg/kg for 42 days had significantly lowered plasma lipid peroxidation and thiobarbituric acid reactive substances (TBARS) levels compared to the untreated diabetic rats [27]. The decreased renal SOD and GSH level in the hypertensive rats increased with the treatment of naringin (40 and 80 mg/kg) for 4 weeks. However, the increased MDA level in naringin treated rats (40 and 80 mg/kg) for 4 weeks was lessened dose-dependently compared to controlled hypertensive rats [28].

## 4. Discussion

This systematic review reveals beneficial findings of naringin in treating hypertension, diabetes, dyslipidemia, and obesity. The findings reveal that naringin is a flavonoid which had been proved to have positive effects on obesity where significant reduction in body weight, liver weight, visceral fat, Lee's index, and abdominal circumference were observed. Apart from that, naringin significantly reduced hypertension by suppressing the systolic and diastolic blood pressures. Furthermore, naringin also reduced glucose level, fasting insulin level by improving *β*-cell function, HbA1c, and hepatic glycogen significantly. Naringin also reduced LDL, TG, TC, NEFA, and VLDL cholesterol in the plasma and liver while significantly reducing the enzyme activity of HMG-CoA and ACAT which are essential enzymes in the production of cholesterol. Moreover, naringin alleviated metabolic syndrome through its radical scavenging ability which prevents radicals from attacking lipids, amino acids, fatty acids, and DNA bases. Lastly, naringin alleviates metabolic syndrome through adipokines and proinflammatory cytokines regulation.

Visceral obesity is defined as excess body weight caused by excessive release of adipokines which eventually increase the level of circulating fatty acids [4]. According to the review, naringin supplementation also reduced abdominal fat deposition, abdominal circumference, body weight, Lee's index, and visceral fat. This might be due to adipocytes apoptosis. Naringin would cause an increase in intracellular calcium, which increases the proteins associated with programmed cell death (calpain and caspase-12) [32]. The apoptosis of adipose cells in adipocytes could aid in weight loss and eventually reduce obesity.

Hypertension is defined as an elevated blood pressure which is a major risk factor for various CVD diseases such as atherosclerosis, coronary artery disease, myocardial infarct, heart failure, and stroke. Studies across the globe have demonstrated that diets rich in plants are able to prevent or treat CVD complications [28]. Naringin as a flavonoid has the ability to be used as antihypertensive drug via its antioxidant potential [28]. Based on the review, naringin suppresses the increase in systolic blood pressure by significantly suppressing the increased level of Nitric oxide (NO) metabolites. NO plays a significant role in the regulation of blood pressure (BP) where decent amount of NO is linked to normal vasodilatation and BP. Thus, a decrease in the levels of NO would lead to failure in smooth muscle relaxation resulting in BP decrease [33]. Moreover, naringin also acts as an antihypertensive potential via its radical scavenging ability by increasing SOD activity and upregulating expression of SOD and GSH in renal [28].

Diabetes or hyperglycemia is a disorder of carbohydrate, fat, and protein metabolism manifested by several abnormalities. The myriad of disorders linked with type 2 diabetes mellitus (T2DM) include insulin resistance, impaired glucose homeostasis, and deficiency of insulin resulting from *β*-cell dysfunction [34]. This systematic review demonstrated that the treatment of diabetic rats with naringin induced beneficial effects through variety of mechanism. Glycosylated haemoglobin (HbA1C) in diabetic rats was increased as a result of excessive circulating blood glucose in the blood. In this review, oral administration of naringin significantly decreased fasting blood glucose and HbA1c concentrations [35]. These results suggest the beneficial effects of naringin in preventing the development of diabetes by decreasing the blood glucose. However, [31] indicated that naringin at dosage of 10 mg/kg for 45 days had no effect on the fasting blood glucose levels. This might be due to the low dosage of naringin (less potent) which could not be able to bind to the receptor to produce the required effect. The present review revealed a highly significant decrease in the fasting insulin level of the untreated diabetic rats due to the destruction of pancreatic *β*-cells. Naringin treatment to the diabetic rats significantly increased the levels of insulin through proliferation of the pancreatic secretion of insulin from *β*-cells and by transport of blood to peripheral tissues. Hepatic glycogen content could be used as a marker for assessing the major symptoms in T2DM. The systematic review revealed the depletion in the hepatic and muscle glycogen content in the diabetic rats which was increased with the treatment with naringin. This is supported by [36] who reported that naringin increased hepatic glycogen storage by regulating gluconeogenesis by lowering the activities of glucose-6-phosphatase and PEPCK. Thus, the utilization of blood glucose would improve and thus decreases the chances of diabetes.

Hyperlipidemia is described as increased circulating lipids in the blood which involves high levels of triglyceride, cholesterol and lipoproteins [37]. Reference [38] indicated that changes in concentrations of plasma lipids including cholesterol and lipoprotein are complications frequently observed in patients with diabetes mellitus and certainly contribute to the development of coronary heart disease (CHD). Flavonoids originated from plants have the ability to lower plasma lipid concentration in the body [39]. In the present review, the increase in TC, LDL, TG, and VLDL and reduction in HDL were observed in obesity induced rats. However, treatment with naringin produced significant improvements of the altered lipid profiles. The decrease of LDL levels may occur due to the reduction of VLDL and the increase of hepatic depuration of LDL precursors [40]. Apart from that, the increase HDL levels may be due to the inhibition of rho-signalling pathways with activation of PPAR-*α* and reduction in cholesterylester transfer protein (CETP) [41]. HMG-CoA reductase, rate limiting enzyme of cholesterol biosynthesis in the liver, apart from lowering plasma total and LDL cholesterol, also lowers plasma triglycerides and VLDL concentrations. In the present review, the bioactivity of HMG-CoA reductase enzyme was significantly decreased in nontreated diabetic rats by the treatment of naringin. This is because naringin may possess statin-liked effect which is one of the lipid lowering agents [42]. Apart from HMG-CoA reductase, ACAT is a cholesterol-esterifying enzyme encoded by two genes that plays important roles in cellular cholesterol homeostasis. In this review, ACAT bioactivity was significantly decreased by naringin treatment. The decrease in ACAT activity is due to naringin which acts as hydrophobic inhibitors that allows them to enter the membrane bilayer such that they can interact with residues that play essential role in substrate binding or in enzyme catalysis [43].

Naringin, apart from alleviating metabolic syndrome directly, also prevents the development of metabolic syndrome through inhibition of oxidative stress and proinflammatory cytokines. Oxidative stress is an imbalance between the production of free radicals (ROS) and the ability of the body to counteract or detoxify their harmful effects through neutralization by antioxidants [44]. Oxidative stress causes lipid peroxidation due to increased ROS leading to overproduction of MDA, which is used as a biomarker [45]. Citrus fruit extract possesses large amounts of flavonoids and shows potent free radical scavenging activity [46]. This statement is supported by [47] who indicated that flavonoids such as naringin and naringenin both are strong scavengers of free radicals and would prevent lipid peroxidation. Based on this review, naringin treatment to diabetic rats significantly raised the concentrations of GST, CAT, SOD, GSH GSH-Px, T-AOC, NO, and GPx in treated diabetic rats. The increase in the level of these endogenous antioxidant defence system is able to trap free radicals generated by cellular metabolism through the donation of hydrogen atom and by breaking the chain reaction, which then prevents the radicals from attack on lipids, amino acids, fatty acids, and DNA bases [48, 49].

Proinflammatory cytokines and adipokines play a critical role in the development of metabolic syndrome. Adipokines such as leptin and adiponectin are secreted by adipose tissues (endocrine organ) which are involved in metabolic regulation and inflammatory processes [50]. Dysregulation in the adipose tissue will induce systemic inflammation and insulin resistance in obese patients [50]. Proinflammatory cytokines such as TNF-*α* and IL-6 are produced primarily from macrophages which increase to account for 40% of total cells in adipose tissue [50]. This review reports significantly elevated levels of proinflammatory cytokines in rats affected with diabetes and obesity. Based on this review, naringin treatment significantly reduced TNF-*α*, IL-6, leptin, and resistin level and significantly increased the level of adiponectin. These results suggest that naringin could reduce or suppress the biological activity and production of the proinflammatory cytokines which in return reduce the inflammation in rats. A summary table was constructed to represent the mechanism of action of naringin in alleviating Mets as shown in Table 2.

There were a few limitations identified from the literatures. The purity of the naringin used in the studies was not indicated except for an article which stated that the purity of naringin used was 88.9%. In addition to that, the source of the plant compound was not stated in any of the studies as they could be from citrus fruit ≥90% or from HPLC ≥95%. The different sources and purity of naringin may produce different outcomes on their effects in suppressing Mets. As a result, replication of these studies will be difficult. This review does not include evidence from humans because supplementation trial in human has not been performed so far. Future studies involving human should consider several aspects of naringin, including its safety, pharmacokinetics, and pharmacodynamic profile. This evidence in humans is lacking to-date. However, with the positive effects in animal and cellular studies, it is worthwhile to continue the efforts of developing naringin as an alternative option to prevent MetS.

## 5. Conclusion

Numerous studies have been done to highlight the role of naringin in alleviating cluster of metabolic diseases. The findings reveal that naringin is a flavonoid which had been proved to have positive effects on hypertension, hyperlipidemia, hyperglycemia, and obesity. However, the dosage used in animal studies might not produce a similar positive effect in human; therefore, currently, dietary recommendation should be given for the intake of pure compound naringin. Thus, adequate large scale of studies should be conducted using oral supplementation at different dosage on different human population to confirm its effect on metabolic syndrome.

## Figures and Tables

**Table 1:** Association between naringin and Mets.

**Tittle of study/year**	**Type of study/ Type of disease**	**Dosage of naringin (mg/kg)**	**Duration**	**Results**
**DPP-IV inhibitory potential of naringin: An *in silico*, *in vitro* and *in vivo* study (2012)**	*In silico, In vitro, In vivo study *& hyperglycemia	40mg/kg	10 days	(i) **In vivo**: Naringin significantly decreased random glucose concentrations, TBARS in pancreatic tissue and non-significantly decreased fasting serum concentrations, pancreatic nitrate concentrations.

**Up-regulation of PPAR**_**y**_ **, heat shock protein-27 and -72 by naringin attenuates insulin resistance, ** **β** **-cell dysfunction, hepatic steatosis and kidney damage in a rat model of type 2 diabetes (2011)**	Animal study & hyperglycemia, hyperlipidemia, oxidative stress,	50 mg/kg & 100mg/kg	28 days	(i) Naringin corrected impaired glucose utilization, insulin insensitivity, reduced *β*-cell function, decreased TC, TAG, LDL, NEFA, significantly increased HDL, repaired injured pancreatic islet cells, decreased TBARS levels and increased SOD, GSH-Px activities in serum/pancreas/liver/kidney, reduced (TNF-*α*), (IL-6), (CRP) and increased adiponectin. (ii) Naringin significantly increased expression of PPAR_y_ in liver & kidney, (P-IRS1) in liver, (HSP-72) & (HSP-27), (NF-KB) in pancreas/liver/kidney.

**Antihyperglycemic and antilipidperoxidative effects of flavonoid naringin in streptozotocin-nicotinamide induced diabetic rats (2010)**	Animal study & hyperglycemia, oxidative stress	80 mg/kg	42 days	(i) Naringin significantly retained the decreased in level of blood glucose with significant increase in level of insulin, significantly lowered TBARS level and normalised the lower levels of plasma GSH, vitamin C and vitamin E.

**Effect of naringin on hemodynamic changes and left ventricular function in renal artery occluded renovascular hypertension in rats. (2014)**	Animal study & hypertension, oxidative stress	80 mg/kg	4 weeks	(i) Naringin significantly increased the heart rate at 15, 30, 45 and 90 mins. (ii) Naringin significantly reinstated systolic and diastolic blood pressure at 15, 30, 45, 60, 90 mins and lessened the mean arterial blood pressure and diastolic pressure (LEVDP) at 15, 30, 45, 60, 90 mins. (iii) Naringin significantly increased renal SOD & GSH and decreased in levels of MDA.

**Preventive effects of hesperidin, glucosyl hesperidin and naringin on hypertension and cerebral thrombosis in stroke-prone spontaneously hypertensive rats (2012)**	Animal study & Hypertension	250 mg/kg, 500mg/kg, 1000mg/kg	4 weeks	(i) Naringin significantly suppressed the increase in systolic blood pressure. (ii) Ingestion of naringin significantly increased the lowered levels NO metabolites.

**Protective effect of rutin and naringin on sperm quality in streptozotocin (STZ) induced type 1 diabetic rats (2010)**	Animal study & Hyperglycemia, oxidative stress	10 mg/kg	46 days	(i) Naringin treatment had no effect on fasting blood glucose levels. (ii) Naringin significantly decreased the elevated levels of MDA, increased the lowered levels of SOD & CAT.

**The citrus flavanone naringin enhances antioxidant status in the albino rat liver treated with doxorubicin (2016)**	Animal study & Oxidative stress	2 mg/kg	NIL	(i) Naringin significantly alter the concentration of SOD and reduced the rate of lipid peroxidation. (ii) Naringin treatment did not significantly alter the GSH, GST, CAT concentrations.

**Naringin reversal of some aspects of diabetic nephropathy in rats type 1 streptozotocin induced diabetes (2015)**	Animal study & hyperglycemia	50 mg/kg	56 days	(i) Naringin significantly improved weight gain, decreased fasting blood glucose, improved plasma insulin secretion, increased hepatic glycogen content, reduced the MDA and increased SOD concentrations.

**Antidiabetic effects of Hesperidin and naringin in type 2 diabetic rats (2012)**	Animal study & hyperglycemia, hyperlipidemia,	50 mg/kg	30 days	(i) Naringin improved the elevated OGTT at 30, 60, 90, 120 mins. (ii) Naringin significantly increased the fasting serum insulin, improved altered level of HbA1c, increased level of HOMA-IR, increased hepatic & muscle glycogen content, reduced TC, TAG, LDL, VLDL, FFA levels, increased HDL levels and reduced the enzyme activity of HMG-CoA and showed significant improvement of serum adiponectin concentrations.

**Upregulation of PPAR**_**y**_ ** mediates the antidiabetic effects of citrus flavonoids in type 2 diabetic rats. (2012)**	Animal study & hyperglycemia	50 mg/kg b.wt.	4 weeks	(i) Treatment with naringin improved OGTT of elevated values at all points. (ii) Naringin significantly improved the altered level of HbA1c, increased serum insulin levels, decreased the elevated levels of HOMA-IR and increased the down-regulation of PPAR_y_, adiponectin levels as well as decreased the resistin levels.

**Naringin improves diet-induced cardiovascular dysfunction and obesity in high carbohydrate, high fat diet-fed rats (2013)**	Animal study & Obesity, hypertension, oxidative stress	100 mg/kg	8 weeks	(i) Naringin reduced retroperitoneal abdominal fat deposition, attenuated the increase in abdominal circumference, reduced plasma lipid concentrations, improved OGTT, normalised the insulin levels and liver weight. (ii) Naringin reduced systolic blood pressure, normalised LVIDd, normalised liver weight, plasma AST, ALT, reduced inflammation and fat droplets.

**The effect of naringin on plasma lipid profile, and liver and intramuscular fat contents of fattening lambs. (2011)**	Animal study & hyperlipidemia	1.5 g/kg	7 weeks	(i) Naringin administration significantly reduced plasma TAG and cholesterol

**Naringin at a nutritional dose modulates expression of genes related to lipid metabolism and inflammation in liver of mice fed a high-fat diet. (2012)**	Animal study & hyperlipidemia, hyperglycemia	100 mg/kg	8 weeks	(i) Naringin significantly increased TC and TG liver contents, normalised insulinemia without modification in glycaemia and HOMA index showed significant improvement in insulin-resistance.

**Naringin ameliorates atherogenic dyslipidemia but not hyperglycemia in rats with type 1 diabetes. (2012)**	Animal study & obesity, hyperlipidemia, hyperglycemia	50 mg/kg	45 days	(i) Naringin treatment improved body mass, lowered plasma LDL, increased plasma HDL, reduced atherogenic index, reduced hepatic TAG & TC levels and the enzyme activity of HMG-CoA & ACAT was reduced. (ii) Naringin did not have a significant effect on the fasting blood glucose and on the glucose intolerance.

**Hesperidin and naringin attenuate hyperglycemia-mediated oxidative stress and proinflammatory cytokine production in high fat fed/streptozotocin-induced type 2 diabetic rats. (2012)**	Animal study & hyperglycemia, oxidative stress	50 mg/kg	4 weeks	(i) Naringin significantly improved the elevated levels blood glucose & HbA1c, increased serum insulin levels, decreased HOMA-IR levels, increased the levels of vitamin C, E & GSH, reduced the elevated levels of TNF-*α* and IL-6.

**Grapefruit derived flavonoid naringin improves ketoacidosis and lipid peroxidation in type 1 diabetes rat model. (2016)**	Animal study & hyperglycemia, oxidative stress,	50 mg/kg	42 days	(i) Naringin significantly improved the weight loss in diabetic rats, increased the reduced hepatic glycogen content, reduced plasma MDA concentration, reduced the elevated levels of serum acetoacetate (AcAc), reduced the levels of serum *β*-hydroxybutyrate (3-HB).

**Naringin mitigates cardiac hypertrophy by reducing oxidative stress and inactivating c-JUN Nuclear Kinase-1 protein in type 1 diabetes. (2016)**	Animal study & hyperglycemia, oxidative stress	50 mg/kg	56 days	(i) Naringin significantly showed reduction in the FBG, increased in plasma insulin, improvement in body weight, reduced plasma and cardiac MDA & carbonyl protein concentrations, increased SOD activity, decreased GPx activity.

**Naringin reduces hyperglycemia-induced cardiac fibrosis by relieving oxidative stress. (2016)**	Animal study & oxidative stress,	50 mg/kg	56 days	(i) Naringin treatment significantly reduced the activity of NADPH oxidase, increased cytosolic SOD, CuZn activity by 65%, reduced in the elevated levels of plasma and cardiac AOPP,

**Naringin ameliorates metabolic syndrome by activating AMP-activated protein kinase in mice fed a high-fat died (2012)**	Animal study & Obesity, hyperlipidemia, hyperglycemia, oxidative stress	200 mg/kg	10 weeks	(i) Naringin significantly reduced Lee's index, liver weight, liver index, visceral fat weight, visceral fat index, weight, decreased fasting blood glucose and reduced serum insulin, decreased the levels of TNF-*α* and leptin, increased levels of adiponectin, decreased LDL and TC and increased HDL significantly. (ii) Naringin significantly decreased liver TC but did not alter liver TG and increased levels of SOD, GSH-Px, CAT, GSH, T-AOC & reduced levels of MDA.

**Table 2:** Summary of naringin action on clusters of Mets.

Naringin and Mets	Mechanism of Action	Outcome
**Effect on visceral obesity**	(i) Increases the intracellular calcium which increases the protein associated with cell death (calpain & caspase-12)	Leads to adipocyte apoptosis and reduce obesity

**Effect on hypertension**	(i) Suppresses the increased level of nitric oxide (NO) (ii) Radical scavenging ability of naringin increased SOD	Leads to failure in smooth muscle relaxation resulting in lower blood pressure

**Effect on hyperglycemia**	(i) Reduced the level of HbA1C and FBG (ii) Increased the level of insulin through proliferation of pancreatic *β* cells. (iii) Increased the hepatic and muscle glycogen content by lowering the activities of G6Pase and PEPCK.	Leads to reduction in circulating blood glucose

**Effect on hyperlipidemia**	(i) Reduced LDL levels by reducing VLDL and increasing the depuration of LDL receptors. (ii) Increased the levels of HDL due to inhibition of rho-signalling pathways with activation of PPAR-*α* and CETP. (iii) Inhibited the activity of HMG-CoA reductase that suppresses the cholesterol homeostasis. (iv) Reduced the ACAT activity by interacting with residues that play essential role in enzyme catalysis.	Leads to reduction in the lipid profiles and cholesterol biosynthesis

**Effect on oxidative stress**	(i) Naringin as a strong radical scavenger prevented the lipid peroxidation by trapping the free radicals through the donation of hydrogen atom and breaking the chain reaction.	Leads to prevention of the radicals attack on lipids, amino acid, fatty acids and DNA bases.

**Effect on proinflammatory cytokines**	(i) Reduced the levels of resistin and increased levels of adiponectin by suppressing the biological activity and production of the cytokines.	Leads to prevention of obesity, insulin resistance and lipid abnormalities
